# UNDERSTANDING THE MEANING OF HOME THROUGH A CO-DESIGNED VIRTUAL PHOTOVOICE STUDY

**DOI:** 10.1093/geroni/igae098.0019

**Published:** 2024-12-31

**Authors:** Joyce Weil, Gitanjali Iyer

**Affiliations:** Towson University, Towson, Maryland, United States; Towson University, Towson, Maryland, United States

## Abstract

Co-designed photovoice is a way for older adults to express what a place means to them from their own point of view using images and narrative. The goal of this study is to understand the concept of “home” using photovoice through the lens of older adults living in Baltimore, Maryland. As part of this co-designed, IRB-approved study, 14 older adults were interviewed twice via Zoom, a video calling platform, about the meaning of home and their community or neighborhood. Participants shared and explained the meaning of 10 photographs regarding what home meant to them. In the second Zoom interview, participants explained the meaning behind each photo and why they selected them. After analyzing the transcripts and photos of each participant, some common themes were found highlighting the meaning of home for the participants. Themes were about symbolism and meaning of objects around their home, neighborhood accessibility and walkability, daily routines and practices, and how “home” had changed during the pandemic. The ways in which virtual co-designed photovoice were based on older adults’ reframing and designing the study will be discussed.

